# Supplementary material for: Integrating genomic structural equation modeling and experimental validation to unravel the genetic basis of male genital lichen sclerosus
Source: Front Immunol. 2026 Jul 16;17:1857945. doi: 10.3389/fimmu.2026.1857945 (PMC13435250; doi:10.3389/fimmu.2026.1857945)
Supplement: Supplementary file 6 [file SupplementaryFile5.zip › result/fuma_PSYCH_factor_sCCA2_focus.chr19.chr19.18409862.19877471.pop1.pdf]

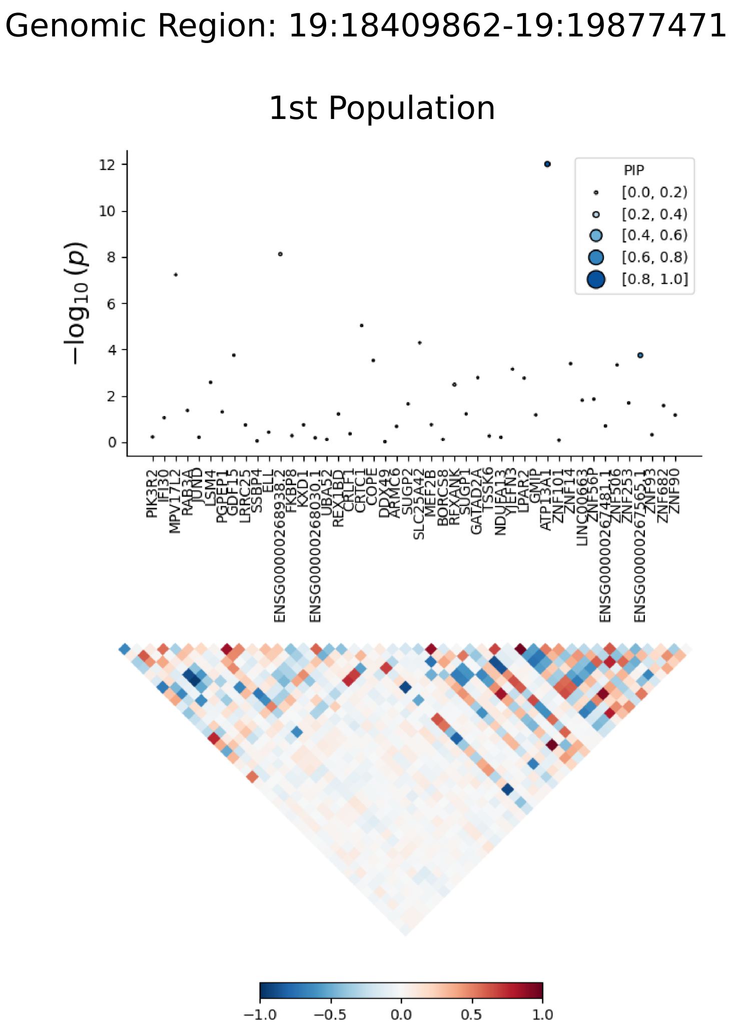
Genomic Region: 19:18409862-19:19877471
1st Population
$-\log_{10}(p)$
PIP
[0.0, 0.2)
[0.2, 0.4)
[0.4, 0.6)
[0.6, 0.8)
[0.8, 1.0]
PIK3R2
IFI30
MPV17L2
RAB3A
JUND
LSM4
PGPEP1
GDF15
LRRC25
SSBP4
ELL
ENSG00000268938.2
FKBP8
KXD1
ENSG00000268030.1
UBA52
REX1BD
CRLF1
CRTC1
COPE
DDX49
ARMC6
SUGP2
SLC25A42
MEF2B
BORCS8
REXANK
SUGP1
GATAD2A
TSSK6
NDUFA13
YJEFN3
LPAR2
GMIP
ATP13A1
ZNF101
ZNF14
LINC00663
ZNF56P
ENSG00000267481.1
ZNF506
ZNF253
ENSG00000267565.1
ZNF93
ZNF682
ZNF90
